# Telehealth and Artificial Intelligence Insights into Healthcare during the COVID-19 Pandemic

**DOI:** 10.3390/healthcare10020385

**Published:** 2022-02-18

**Authors:** Dina M. El-Sherif, Mohamed Abouzid, Mohamed Tarek Elzarif, Alhassan Ali Ahmed, Ashwag Albakri, Mohammed M. Alshehri

**Affiliations:** 1 National Institute of Oceanography and Fisheries (NIOF), Cairo 11516, Egypt; 2 Department of Physical Pharmacy and Pharmacokinetics, Poznan University of Medical Sciences, 60-781 Poznan, Poland; mmahmoud@ump.edu.pl; 3 Doctoral School, Poznan University of Medical Sciences, 60-781 Poznan, Poland; alhassan.ahmed@student.ump.edu.pl; 4 Independent Digital Health Researcher and Entrepreneur, CEO Doctor Live Company, Cairo 12655, Egypt; muhammad.elzarif@gmail.com; 5 Department of Bioinformatics and Computational Biology, Poznan University of Medical Sciences, 60-781 Poznan, Poland; 6 Collage of Computer Science and Information Technology, Jazan University, Jizan 45142, Saudi Arabia; aoalbakri@jazanu.edu.sa; 7 Medical Research Center, Jazan University, Jizan 45142, Saudi Arabia; moalshehri@jazanu.edu.sa; 8 Physical Therapy Department, Jazan University, Jizan 82412, Saudi Arabia

**Keywords:** COVID-19, healthcare, digital health, pandemic, telemedicine, artificial intelligence, telehealth

## Abstract

Soon after the coronavirus disease 2019 pandemic was proclaimed, digital health services were widely adopted to respond to this public health emergency, including comprehensive monitoring technologies, telehealth, creative diagnostic, and therapeutic decision-making methods. The World Health Organization suggested that artificial intelligence might be a valuable way of dealing with the crisis. Artificial intelligence is an essential technology of the fourth industrial revolution that is a critical nonmedical intervention for overcoming the present global health crisis, developing next-generation pandemic preparation, and regaining resilience. While artificial intelligence has much potential, it raises fundamental privacy, transparency, and safety concerns. This study seeks to address these issues and looks forward to an intelligent healthcare future based on best practices and lessons learned by employing telehealth and artificial intelligence during the COVID-19 pandemic.

## 1. Introduction

The coronavirus disease (COVID-19) pandemic has affected the environment, and people’s health lifestyle globally [1,2]. Digital health offers a valuable opportunity to handle epidemics such that real-time results continuously emerge. Recent cases of Severe Acute Respiratory Syndrome (SARS), influenza A virus subtype H1N1, and Ebola Virus Disease have taught us many lessons about the usefulness of digital health in public health crises. Those lessons can also be applied to improve our reaction to the coronavirus disease 2019 (COVID-19) pandemic caused by severe acute respiratory syndrome coronavirus 2 (SARS-CoV-2) through innovative and productive techniques [3,4,5]. In 1980, the Veterans Health Information Systems and Technology Architecture (VisTA) was deployed for the first time; this is considered the beginning of what is now referred to as digital medicine, leading to the first generation of the Electronic Health Record (EHR). The successful implementation of the computerized patient record system was another milestone in 2000. The VisTA user interface allows providers to analyze and edit the EHR of patients, which was the beginning of medical information technology [6].

The launch of the first iPhone in 2007 contributed to developing an ecosystem that enables real-world monitoring and clinical/research-level health data collection through mobile systems. Today’s social, mobile, computational, and cloud integration creates a society of customers immersed in technology. The third technological revolution (digital technology) has already taken place and has given rise to significant developments in the medical world, but the subsequent fourth medical revolution will have a significant impact on healthcare [7,8,9]. The fourth medical revolution began with technologies such as the Medical Internet of Things (MIoT), artificial intelligence (AI), advanced robots, biosensors, etc. These innovations have digitized services for the medical sector to enhance healthcare services [10,11,12]. Hence, the goal of the fourth medical revolution is to plan and build an intelligent healthcare system to function effectively and efficiently and create a better technology platform for virtualization, decision making, and real-time capability [13,14]. The principal promoting innovations of the fourth medical revolution are highlighted in Table 1 [15,16,17,18,19,20,21].

Telehealth has a broader scope of remote healthcare services than telemedicine. Different terminologies are used to refer to telemedicine or telehealth—for instance, digital health, electronic health, mHealth (Mobile Health), teleconsultation, and tele-triage. In addition, there are some terms that mention specialties, e.g., teleneurology, telecardiology, and telepsychiatry [22]. Telemedicine has been used since the early 1960s by the military and space technology departments. Nowadays, telemedicine is available for everyone at digital stores, and mHealth apps can be used on smartphones, tablets, and computers [23]. These apps provide accessible remote communication between healthcare professionals and their patients [24].

Telemedicine has some disadvantages, such as the need for network stability, good battery life, data security, and privacy. Some critics of telemedicine argue that using these apps is unethical in terms of privacy, equity, and patients’ rights [24,25]. Additionally, mHealth apps may increase the spread of inaccurate information due to the absence of face-to-face communication. A limited number of studies have reviewed this issue, but it remains a safety concern that should be addressed [26,27]. This study investigates the role of telehealth and AI in combating the COVID-19 outbreak through identifying hotspots in digital health during COVID-19. We also report data privacy and security challenges that researchers must be aware of and explore the available tools and techniques to minimize the risks.

## 2. Bibliometric Analysis Methodology

### 2.1. Source and Search Query

The bibliometric method was based on the Clarivate Analytics online subscription database Web of Science™ (WOS) to search the literature. WOS permits a comprehensive investigation of major fields or subfields, as previously reported [28,29]. In this study, we adjusted the document type to “article,” the language to “English,” and the period from 2019 to December 2021. Field tag, “TI = Title; and AK = author keywords” were utilized in the search strategy, and the following query was used:

(TI = (Digital health AND COVID-19)) OR AK = (Digital health AND COVID-19).

We built the bibliometric networks using VOSviewer (developed by Nees Jan van Eck and Ludo Waltman, Centre for Science and Technology Studies, Leiden University, The Netherlands) [30].

### 2.2. Bibliometric Maps of Co-Citations

**Figure 1:**
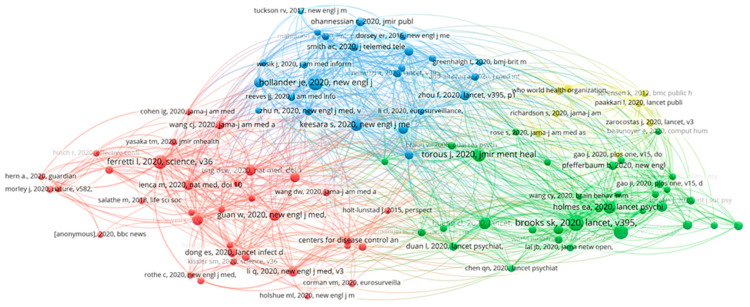
Co-citation map among COVID-19- and DH-related articles.

This feature identifies multiple references that the same publication has cited. Given a total of 16,680 cited references, the minimum citation count was set to five; only 100 met the criteria and were designated for visualization (Figure 1).

### 2.3. Hotspots of Papers Related to Digital Health

**Figure 2:**
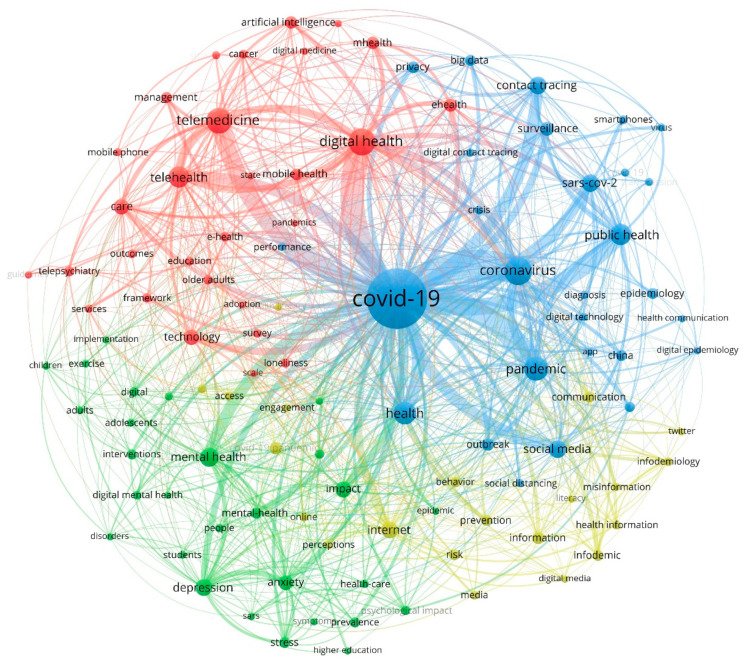
Top trending keywords related to DH and COVID-19.

Keywords related to digital health and COVID-19 were analyzed by VOSviewer and shown in Figure 2. The keyword map was designed by selecting authors and keywords mentioned at least five times. Out of 2125 keywords, 106 were selected and categorized into four clusters.

## 3. Results

### 3.1. Source and Search Query

The extracted results (468 publications) were analyzed further using VOSviewer, and the top five categories (healthcare sciences services, medical informatics, public environmental, occupational health, psychiatry, and general internal medicine) accounted for over 70% of publications. 

### 3.2. Bibliometric Maps of Co-Citations

Regarding bibliometric maps of co-citations, four clusters were created. The first cluster (red) had 34 publications, and is focused on quantifying SARS-CoV-2, isolation, and digital contact tracing. The second cluster (green) encompassed 33 publications, and the central topic discussed was the psychological responses and digital mental health. The third cluster (blue) encompassed 24 publications and primarily investigated telemedicine for COVID-19. Digital education in COVID-19 and COVID-19 mortality statistics were the fourth cluster’s (yellow) main topics, with eight publications.

### 3.3. Hotspots of Papers Related to Digital Health

Concerning the hotspots analysis, cluster 1 comprised 29 keywords; its main focus was the management of community and induvial healthcare through digital health, telemedicine, telehealth, and artificial intelligence. Cluster 2 consisted of 28 keywords regarding mental health issues, anxiety, and stress during COVID-19 and how to cope with them. Cluster 3 consisted of 28 keywords; its main topic was using big data in surveillance studies, including tracing apps and their privacy concerns. Cluster 4 consisted of 21 keywords related to population engagement on the internet and social media and sharing information and its validity.

Only keywords from the first cluster of the hotspots were included for further discussion. Through the third cluster in the bibliometric map of co-citations, commercial search engines, and local directories, we were able to identify telehealthcare technologies.

## 4. Discussion

### 4.1. Telemedicine, Telehealth, and Mobile Health (mHealth)

Telemedicine enables clinical services to use information technology, video imaging, and telecommunication links to deliver healthcare services at a distance. In contrast to telemedicine, which is defined as the provision of medical services at a distance by a physician, telehealth is an umbrella word that encompasses telemedicine as well as a number of nonphysician services such as telenursing and telepharmacy [31].

Telemedicine is often used for controlling chronic diseases such as cardiovascular diseases, diabetes mellitus, cancer, and mental disorders. Telemedicine might be a safe and effective alternative for older people who suffer from these diseases. It is easy for patients to follow up on their cases via mHealth apps, especially those living in rural areas [32].

Digital psychotherapy is considered one of the most successful roles of telehealth. It makes it easy for patients to communicate with their psychiatrists anytime and anywhere. Telepsychiatry costs less than regular visits to therapists. Due to the severe shortage of mental health professionals in rural areas, digital psychotherapy has developed to help people in the countryside communicate with their psychiatrists in urban areas [33]. Mobile apps could be an effective alternative to telepsychiatry services for patients. People with depression, anxiety, schizophrenia, and other mental illnesses can benefit from technology and be cared for at home using their smartphones [34].

Cancer is the leading cause of death worldwide. Most cancer patients need regular monitoring to control their health. Cancer is a chronic disease, and the patient’s family has a vital role in improving the patient’s quality of life. The family’s contribution to the patient’s care at home is essential. Palliative care programs are based on the family’s responsibility for care at home. Family members face challenges providing care at home, and telemedicine provides them with information and knowledge. Mobile apps can facilitate communication between cancer patients and their healthcare providers [35]. Table 2 presents examples of telehealth apps around the world.

### 4.2. Telehealthcare’s Role during COVID-19 Pandemic

The importance of telemedicine has garnered more attention since the COVID-19 pandemic. Teleconsultation is a safe and effective way to diagnose, control, and treat diseases [15]. Suspected COVID-19 cases or infected patients (with mild and moderate cases) are advised to stay at home and use mobile apps to follow up with their healthcare providers. Many governments launched telehealth apps to provide online health services for citizens. The Brazilian Board of Medicine published a memorandum on 19 March 2020 to use telemedicine as an exception during the pandemic [58]. The MOH (Ministry of Health) of the Republic of Indonesia encouraged telehealth services for COVID-19 related inquiries or any other medical conditions. The MOH prompted health-tech start-ups to release mobile apps that provide digital health services [48]. In Turkey, Syrian refugees suffer from low quality of life, low socio-economic status, language challenges, and poor health conditions. Hence, telemedicine services are a cost-effective alternative for those refugees to contact healthcare practitioners in Arabic and English [49]. The Indian Space Research Organization, MOH, and Ministry of External Affairs played a significant role in developing telemedicine services in India. The government supports and promotes telemedicine during the COVID-19 pandemic to reduce overcrowding in hospitals and encourage social distancing. During the COVID-19 pandemic, telemedicine can also help with reducing the burden on tertiary hospitals by providing diagnosis and treatment to patients in their own location and reducing chances of the patient’s exposure due to hospital visits [52].

### 4.3. Artificial Intelligence in the Healthcare Sector

#### 4.3.1. AI Types and Subgroups

The term AI was introduced to the world for the first time by McCarthy et al. in the 1950s [59]. The term AI refers to the ability of computer systems to think and take action like humans in comparable situations and predict the outcomes of these reactions. The AI-based algorithms continue to be improved by developers and scientists, taking advantage of the refinement of networks and technology infrastructures, especially in the late 1990s. AI has been classified into seven types based on functionality and technology (Table 3). AI has two related subgroups: machine learning and deep learning. AI refers to intelligent systems that think and act like humans. Machine learning refers to systems that learn things based on previous experience and provide defined data to make proper decisions, while deep learning refers to systems that can think like human brains using artificial neural networks [60].

#### 4.3.2. AI Applications

Nowadays, AI has become a useful tool for users in many different fields, including e-commerce (personalized and online shopping), navigation (GPS technology, traffic prediction), robotics (robots powered by AI), healthcare (diagnosis and prognosis of different diseases, and finding the appropriate treatment approach for each case), agriculture (identify defects and nutrient deficiencies in the soil), gaming (creating human-like interactions and predict the human behavior), automobiles (self-driving vehicles), social media (Facebook, Instagram, and Twitter), marketing (delivering targeted, personalized ads), and smartphones (facial recognition) [68].

The spatial distribution of diseases was identified and analyzed by Geospatial Artificial Intelligence (GeoAI). It was used to simulate or predict diseases and track them in research on infectious diseases. Google Flu Trends used big spatial data (weekly forecasts for various cities) from the National Climate Data Centre [69], and deep learning recurrent neural networks (RNNs) were used for predicting influenza outbreaks at provincial and city spatial scales in the USA, assisted by bioinformatics tools like docking and modulation to predict upcoming influenza subtypes that could cause a future pandemic [70]. Using an algorithm tailored to artificial neural networks, geotagged tweets from Twitter and the Centers for Disease Control and Prevention and influenza-like illness datasets were also used to forecast illness in real time [71]. These geotagged tweets focused on where the user sent the tweet from, and allowed for its geographical position to be monitored on the Twitter App. Another study used a machine learning approach to predict the epidemiology of influenza in the USA each season, integrating a predictive method of self-correction with Google Patterns relevant to influenza, cloud-based EHRs, and historical flu trends, in addition to a network-based approach that leverages spatiotemporal trends in historical influenza activity [72].

There are currently various GeoAI strategies for public health uses, and broad attempts to deploy GeoAI and location-based information in precision medicine, such as through mHealth for therapies. Future research will broaden current GeoAI technology to open up new opportunities for research and development in the field of spatial epidemiology and public health, including modeling sites that have not already been documented in high resolution, or analytics for the creation of new spatially extensive data sources [73].

#### 4.3.3. Artificial Intelligence’s Role in COVID-19 Prediction

In hospital emergency departments, COVID-19 patients are in a highly critical situation that requires quick interpretation of symptoms so that physicians can make appropriate decisions. AI-based models have been used to predict the danger of deterioration in COVID-19 patients using the X-ray images of their chests and based on artificial neural networks that are fundamental to the deep learning-based algorithms [74]. The AI-based model can learn from physicians’ daily reports, and the trained model used data from 3661 patients. The obtained results, which showed an accuracy of 0.786 of the area under the curve (AUC), could be vital in assisting physicians with diagnosis and reporting findings in the emergency department. Another AI-based model was designed to see how well a chest radiograph performs through scoring of the severity of COVID-19. The model was integrated with laboratory and clinical evidence to predict the outcomes of COVID-19 for infected patients [75]. The obtained results showed an accuracy of 84% and AUC = 0.82. Two radiologists evaluated the results, confirming the accuracy of the AI-based model findings, which will assist radiologists with chest radiograph reports and predicting COVID-19 patient outcomes in the future.

Moreover, a machine learning-based model has been designed to predict the risk of infection by SARS-CoV-2. The model was tested and trained using data from more than 51,500 patients who were diagnosed with COVID-19. The designed model used eight features collected from the COVID-19 patients, including age, sex, confirmed contact with an infected individual, and five clinical symptoms (cough, fever, sore throat, shortness of breath, and headache). Based on these features, the obtained results showed AUC accuracy with 95% CI: 0.892–0.905 [76]. Overall, the AI models and algorithms had a vital role during the COVID-19 pandemic, as users trust them to make proper decisions about diagnosis and infection outcomes and assist physicians throughout the reporting process of to achieve suitable and quick intervention.

### 4.4. Future Perspectives and Potential Challenges Facing Mobile Technologies and Data Sharing in Health and Healthcare

Technologies such as the Internet of Services provide new possibilities in the medical field, reducing operation times and risks. Technology enables better knowledge of COVID-19 infection levels. Doctors can anticipate patient outcomes following therapy. These technologies are intelligent enough to solve a variety of issues in healthcare, particularly detection, process simulation, analysis, and therapy selection. Virtual healthcare consultations will become readily available in the coming days, reducing the need for face-to-face contact. Doctors can use Internet-connected technologies to digitally monitor their patients in remote regions and address numerous ongoing issues of the COVID-19 pandemic, bringing about a new era in medicine. The monitoring systems provide for patient care in times of emergency [21].

Telemedicine has a lot of potential benefits, but also many disadvantages [21]. Telemedicine’s main drawbacks include a breakdown in the interface between health professionals and their patients, a breakdown in the relationship between health professionals, issues with the quality of health data, and organizational and bureaucratic challenges [77].

Mobile technology, cloud storage, broadband networking, and wearable devices are increasingly embraced by researchers, experts, and consumers alike, effectively removing the traditional boundaries around sensitive data. Consequently, procedures to secure this information must be implemented at the source. Healthcare professionals and academics are now operating in an Internet-enabled digital ecosystem of technologies that are loosely coupled, easy to install, and offer effective care delivery and measurement capabilities. However, there are enormous difficulties involved in maintaining the privacy and confidentiality of individuals and their records [6].

Contextual [78] and ethical [79] issues have been raised about data sharing, including a lack of standardized privacy protections. We need ways to enhance health data exchange and connection and create consensus on data governance [80]. Comprehensive legislation like the General Data Protection Regulation in the EU has started to tackle this issue, but more tailor-made methods, such as the voluntary privacy code for mHealth apps [81], are needed. Recent ethics studies [82] also found that consumers need to consider and agree to all facets of the use of their results.

Obstacles noted for data exchanged in Africa are a lack of control as soon as the knowledge is shared; suboptimal benefits for producers or administrators of information; unreasonable advantages arising from a more advanced technological background; and technical questions related to data consistency, interoperability, and misinterpretation risks. Many of the technological challenges are known to have largely been resolved. Problems related to anxiety, risk, and insecurity in places such as Africa where data sharing may not be optimized are less recorded. Ethical implications may also be less often discussed or dealt with in these countries [78].

Official data-sharing standards are occasionally missing, unclear, or inconsistent. The balance between making data available, maintaining the privacy, and protecting public health staff’s intellectual, time, and financial contributions are not always well controlled or defined, resulting in protective policies on public health data sharing in general [83].

Public health science advocates continue to urge reform and create strategies for data sharing. However, the barriers behind the lack of data sharing have not yet been adequately discussed. This includes systems that reward research but not data publishing, a lack of funding, and job pathways that underestimate vital data processing practice. Practical problems must also be resolved: how and where can long-term data be kept, who manages access, and who pays for these services? Current guidelines on metadata must be loosened to make health information easily accessible [80].

## 5. Conclusions

Pandemics have proven the importance of digital health as an integrated part of public health, especially when social distancing is required or the number of patients is high enough to overwhelm a medical facility. AI can combat COVID-19; AI-assisted detection is safer, more accurate, and faster than previous methods. Protein structure prediction, therapy monitoring, awareness, social control, and digital health are all recognized applications of AI in the fight against COVID-19. Also, telehealth has the potential to play a critical role in the pandemic planning and response. It offers numerous benefits during a pandemic, such as expanding access to healthcare, reducing disease exposure for staff and patients, preserving scarce supplies of personal protective equipment, and reducing the patient demand on facilities. Still, privacy and security are major challenges for governments and policymakers to ensure the safe development and deployment of AI and telehealth for the public. Finally, the authors propose an action plan to enhance data sharing methods that will benefit all stakeholders, including data collectors, analysts, policymakers, and, eventually, the general public.

## Figures and Tables

**Table 1:** The principal promoting innovations for fourth medical revolution.

Technology	Feature
Telemedicine	This technology is useful for ensuring social distancing during the COVID-19 pandemic. It is beneficial for medical care in rural areas to use telecommunication technologies.
Biosensors	Biosensors can be used to distinguish signs of viral infection related to COVID-19. Useful to measure temperature and related changes in the patient’s symptoms. Perform the necessary functions as per the sensor’s design in any crisis.
MIoT	MIoT connects all healthcare tools and devices to the Internet to access real-time information. It gives greater control of chronic conditions.
Robotics	This technology is used in the hospital to scan an infected patient. Scan all patients, healthcare staff, and guests, with minimal risk to the doctors. Provide critical and noncritical services in remote and challenging locations.
Cloud storage platform	The platform uses computers to store data in cloud storage. All information is stored remotely after the care of the infected patient, which helps to achieve improved outcomes in the future.
Data security	Data management guarantees the security of medical data. Improves the administration of various therapy programs. Helpful in preserving and tracking confidential information about the transmission of this outbreak, inventory of services, medications, etc.
AI	AI with adequate preparation has a human-like intellect that helps anticipate, monitor, and interpret signs of COVID-19 or related diseases. It analyzes signs of cold, cough, fever, and other symptoms in patients with COVID-19. Enables support for telemedicine and provides tracking for individuals and clusters. Can be built into other systems and technologies for predictive analysis and disease modeling. Has implementation in the manufacture of drugs/vaccines. Helpful to classify the mortality rate and other anomalies of disease data.
Analytics of big data	The entire medical history of all patients is digitally, and anomalies are further examined. stored Rapidly recognize any signs of the given virus.
Bioactuators	Provides essential functions for the patient’s bed, operating table, and chair. Raise and lower bed according to the patient’s needs during treatment.
Blockchain	Pandemic control system algorithm for COVID-19. Applicable during a crisis for patient track and trace and disease/infection prevention. Involves outlining in the early stages of the disease.
Information technology	Provides a considerable change in information and data processing. Helps with the availability of backbone hardware and software for care.
Imaging technologies	Increased reality (AR)/virtual reality (VR) systems will promote learning and training in remote locations. AR/VR images can be used by a remote specialist in patients with major defects and recommended treatment. Health apps can encourage increased patient satisfaction.
Additive manufacturing	Consists of 3D scanning, 3D printing, and other software design and printing. 3D scanning is helpful to construct the necessary design for a part of the patient. 3D/4D/5D printing consumes less time and money, and satisfies the scarcity of critical COVID-19 pandemic items. The optimized design could be researched, evaluated, and improved.
Nanomedicine	Aid in healing infected patients’ cells with the help of protein repair. Nanoparticles the size of the novel coronavirus can be successfully used in patient care. Has the ability to cure and control infections in this ongoing scenario.

**Table 2:** A summary of telehealthcare technologies around the world.

App Name	Country	Launch Date	Description
Amwell	USA	June 2006	Amwell is a telehealth platform that offers telemedicine services for healthcare providers and their patients [36].
Zocdoc	USA	April 2007	Zocdoc is a technology company that provides an online appointment scheduling platform [37].
Ping An Good Doctor	China	April 2015	A mobile platform for online consultations, hospital referrals, and appointments (Internet hospital) providing online healthcare services [38].
Babylon	UK	January 2013	Babylon is a teleconsultation app that provides many services on behalf of general practice in London [39].
KRY	Sweden	April 2014	KRY is a health tech company that provides consultations via smartphone instead of conventional face-to-face appointments for primary care [39].
Doctor Anywhere	Singapore	2016	Doctor Anywhere is a digital platform offering quick access to health services [40].
Qare	France	2016	Qare is a platform that provides medical video consultation services [41,42].
Videodoc	Ireland	July 2014	VideoDoc is a healthcare and medical service that offers scaled access to online healthcare services [43,44].
Okadoc	UAE	2018	Okadoc is an online appointment booking platform to connect healthcare professionals with their patients [45].
MediQuo	Spain	2017	MediQuo is a 24/7 medical chat app for healthcare providers and their patients [41].
Maple	Canada	July 2015	Maple is a virtual care platform. Through Maple, you can speak with doctors through text or video, receive diagnosis and prescriptions. [46].
TeleClinic	Germany	2015	TeleClinic is a telemedicine platform that enables simple and secure communication between healthcare providers and patients [47].
SehatPedia	Indonesia	February 2019	The Indonesian MOH launched SehatPedia App to facilitate access to healthcare providers [48].
HERA	Turkey	2018	The HERA App is a health platform for the Syrian refugee population in Turkey. It provides services in the three most commonly spoken local languages: Arabic, Turkish, and English [49].
Little Dot	Croatia	2006	Little Dot is a health platform for remote video consultations from the user’s home [50].
Al-Sehha	Saudi Arabia	Dec. 2017	The Saudi Arabian MOH created the Al-Sehha mobile app. It provides e-consultations in audio and video modes for users at home [51].
Practo	India	2008	Practo is a mHealth platform that provides access to a vast network of doctors and clinics in India. It connects patients with healthcare providers through calls or chats [52].
Yandex Health	Russia	November 2016	Yandex Health is an online consultation with doctors (all specialists) [53].
Instant Consult	Australia	2017	Instant Consult connects doctors with patients instantly for online health consultations via video call [54].
Vezeeta	Egypt	February 2012	Vezeeta is a digital healthcare platform that provides teleconsultations and booking services for private clinics [55].
Udok	South Africa	2018	Udok ia developed a telehealthcare system to connect medical practitioners with patients remotely [56].
Health Connect	Nigeria	2018	Health Connect is a telemedicine app [57].

**Table 3:** Functionality- and technology-based classification of AI.

AI Type	Properties	Ref.
Functionality based		
Reactive Machines	The earliest AI type, with the most limited technological capabilities. Operate without the need to have a memory base. Mimic the human brain’s capabilities to react to many sorts of scenarios. Cannot store experiences and use the results to predict future responses.	[61]
Limited Memory	It merges the ability to react and learn from previous information. Has many learning algorithms; the collection of datasets helps AI systems with analyzing current situations and learning from past experiences to make decisions in the future. The best example of AI-limited memory systems is the fingerprint scanning machine.	[62]
Theory of Mind	It is designed to perform a broad range of analyses—of thought patterns, emotions, and belief systems. It will help robots better comprehend people and understand the different elements that impact their thinking.	[63]
Self-Aware	Theoretical systems remain under development. Sophisticated systems comparable to the human mind; will have self-consciousness and be self-sufficient. It is still uncertain how long these AI systems will take to evolve.	[64]
Technology-based		
Artificial Narrow Intelligence (ANI)	Now, ANI is the most widely used category of AI. ANI is capable of doing one or two jobs at the same time. It takes advantage of the training data, and experiences gained from past situations. Currently, all models of AI may be classified as a part of ANI, from fundamental methods to advanced algorithms used by computers to make decisions. ANI is considered a weak AI system because it operates within a limited set of parameters	[65]
Artificial General Intelligence	This is linked to the Theory of Mind for functionality scientists are still working on. The goal is to design devices that can independently create connections throughout several domains.	[66]
Artificial Super Intelligence (ASI)	It is designed to execute activities and make choices better than humans, who demand massive quantities of memory. Machines with a large amount of memory, a higher processing speed, and a faster rate of intelligent decision-making would be able to perform complicated tasks with ease and in less time. They will be able to make complex decisions impacted by various circumstances in ways they have never been able to before. It is still under development.	[67].
